# Towards a Spatial Understanding of Trade-Offs in Sustainable Development: A Meso-Scale Analysis of the Nexus between Land Use, Poverty, and Environment in the Lao PDR

**DOI:** 10.1371/journal.pone.0133418

**Published:** 2015-07-28

**Authors:** Peter Messerli, Christoph Bader, Cornelia Hett, Michael Epprecht, Andreas Heinimann

**Affiliations:** 1 Centre for Development and Environment, University of Bern, Bern, Switzerland; 2 Institute of Geography, University of Bern, Bern, Switzerland; 3 Centre for Development and Environment, Lao country office, Vientiane, Lao PDR; National University of Singapore, SINGAPORE

## Abstract

In land systems, equitably managing trade-offs between planetary boundaries and human development needs represents a grand challenge in sustainability oriented initiatives. Informing such initiatives requires knowledge about the nexus between land use, poverty, and environment. This paper presents results from Lao PDR, where we combined nationwide spatial data on land use types and the environmental state of landscapes with village-level poverty indicators. Our analysis reveals two general but contrasting trends. First, landscapes with paddy or permanent agriculture allow a greater number of people to live in less poverty but come at the price of a decrease in natural vegetation cover. Second, people practising extensive swidden agriculture and living in intact environments are often better off than people in degraded paddy or permanent agriculture. As poverty rates within different landscape types vary more than between landscape types, we cannot stipulate a land use–poverty–environment nexus. However, the distinct spatial patterns or configurations of these rates point to other important factors at play. Drawing on ethnicity as a proximate factor for endogenous development potentials and accessibility as a proximate factor for external influences, we further explore these linkages. Ethnicity is strongly related to poverty in all land use types almost independently of accessibility, implying that social distance outweighs geographic or physical distance. In turn, accessibility, almost a precondition for poverty alleviation, is mainly beneficial to ethnic majority groups and people living in paddy or permanent agriculture. These groups are able to translate improved accessibility into poverty alleviation. Our results show that the concurrence of external influences with local—highly contextual—development potentials is key to shaping outcomes of the land use–poverty–environment nexus. By addressing such leverage points, these findings help guide more effective development interventions. At the same time, they point to the need in land change science to better integrate the understanding of place-based land indicators with process-based drivers of land use change.

## Introduction

In the debate about future Sustainable Development Goals, natural scientists have outlined environmental planetary boundaries delineating what they refer to as a “safe living space for humanity” [1]. In response, civil society organizations took the lead in responding that this environmental ceiling must be complemented with social foundations (e.g. food security, gender equality, and access to education), below which lie many dimensions of human deprivation. Accepting planetary boundaries as an outer boundary for human development but combining it with social foundations as an inner boundary, they proposed a doughnut-shaped area considered to represent an “environmentally safe and socially just space for humanity to thrive in” [2]. If this safe and just living space is to guide future development, any policy striving for sustainable development—be it at the local, national or even global level—will essentially need to navigate trade-offs and inherent conflicts between competing interests and claims on future development, between different sectors of the social, economic and environmental realm, and between different subsystems and scales of the earth system [3]. Mitigating such conflicts means navigating trade-offs equitably and transparently, in turn requiring that decisions and policies be informed by the best available evidence. Thus, sustainable development oriented research will not only need to reveal sustainability trade-offs across scale, time, and space, but also winners and losers—and it will have to explore transformations to alternative development pathways [4,5].

A prominent field addressing such research questions is land change science. Land change science studies the dynamics of socioecological systems from a sustainability perspective [6,7]. Focusing on processes and activities related to the human use of land, it analyses benefits gained from land as well as the social and ecological outcomes of societal activities [8]. But land change science still faces a key challenge: producing generalized knowledge on land-based socioecological interactions that goes beyond local cases—and coupling this with global and regional driving forces of land change [9,10]. This challenge is clearly reflected in the heterogeneous and often contradictory body of knowledge on the nexus of land use, poverty, and environmental degradation.

The guiding research hypothesis stipulates the existence of mutually reinforcing links between poverty and environment mediated through land use. The search for general theories about these links has engaged researchers since colonial times, leading to three distinct but closely related arguments [11]. First, poor people are considered more likely to degrade the environment, because households with limited livelihood options deplete resources for their short-term needs. Given the growing number of rural poor in the global South, the argument that more poor people will further burden natural resources is taken up by studies explicitly or implicitly rooted in neo-Malthusian understandings [12]. This simplification is used in most global and regional models assessing the expected consequences of raising food demands and agricultural expansion [13–15]. Second, with the emergence of the concept of sustainable development, poverty and environmental degradation were increasingly seen as a vicious circle in which poor people, particularly dependent on ecosystem services, accelerate environmental degradation [16,17]. Third, it is argued that the vicious circle can turn virtuous when policies to alleviate poverty and protect the environment are integrated. This integration helped to appease the tensions between social and environmental goals, especially in nations of the South, and gave rise to many integrated development and conservation programmes. Yet, critics suggest that this turn obscured the inherent trade-offs between development and environment. Rather than revealing and addressing competing interests hindering sustainable development, a narrow focus on assumed synergies allowed neo-liberal economic agendas to co-opt environment and development thinking and actually aggravate non-sustainable development trends [11,18].

Nevertheless, insights emerging from specific case study research have revealed the weaknesses of generalized hypotheses on the nexus between land use, poverty, and environment. These studies show that the definition of poverty must be related to a concrete local context [19] and that poverty–environment relations can only be understood by scrutinizing factors that determine resource access, including endowments and entitlements [20]. Multiple and nested institutions also play a key role in regulating local access to, and use of, natural resources [21–23]. Increased pressure on land may also lead to intensified land use and improved environmental stewardship [24], or conversely, poor people may adapt successfully to environmental change [25]. Finally, critics point to the methodological challenge of accurately relating changes in human well-being to environmental degradation, given the growing disconnection across scale and geographical distance [26,27].

In summary, it is generally agreed that changes in human well-being and the environment, that are related to changes in land use, are found to be highly complex and often context specific. It is therefore very difficult to make generalizations about dynamics at broader scales and in different places [28,29]. This limited validity of research findings has been referred to as the “one place–one time syndrome” [30]. It represents a persistent obstacle to informed policy and decision-making which increasingly rely on generalizations applicable to various levels of administrative scale beyond the local context.

The Lao Peoples’ Democratic Republic (Laos), a landlocked country in Southeast Asia, represents a powerful example of how development policies are increasingly shaped by national or global influences and largely ignore the differences in local land systems. While the country’s abundant land resources represent an important development asset, poverty and inequality are still widespread [31]. As the mainly natural-resource based economy, driven by regional and global economic integration, grows by approximately 8% [32], trade-offs between the use of land, environmental protection, and poverty alleviation become increasingly evident [33–35]. Various reports from the field describe the impacts of policy decisions and related trade-offs: foreign direct investments into hydropower, mining, and large-scale agribusinesses lead to fierce competition over land, with local people being evicted from their villages and land [35–37]. Traditional shifting cultivation, or swidden agriculture, as we will refer to it here, still provides food security for large parts of the rural population, while at national level it is blamed for deforestation, loss of biodiversity, and increased carbon emissions [34,38,39]. Fallow and communal lands, protected only by weak tenure rights, represent a preferred target for large-scale land acquisitions or the extension of protected areas [37]. This loss of resource access of local populations gives rise to new forms of poverty [40]. Finally, evidence has emerged that intensifying agriculture may also lead to biodiversity loss and increased livelihood vulnerability [41–43].

These examples demonstrate the ambiguity of decisions and policies made at provincial, national, or even international level. Do they just highlight short-term, unavoidable trade-offs to an economic development that is beneficial in the long-term? Or do national growth strategies systematically increase disparities, create new forms of poverty, and exploit the natural resource base? Hence, what is the price of land use modernization and intensification in terms of food security, social cohesion, and environmental degradation? Many of these questions remain unanswered. Knowledge that aims at making policy decisions more evidence based must be able to overcome the gap between the need for generalization at higher or macro-levels of spatial scale and the requirement to account for specificities of different development contexts at local or micro-level. Various approaches have been developed in land change science to overcome this gap. Meta-analysis of case studies has gained increasing attention, as it allows single case studies to be contextualized and may reveal recurrent patterns of different drivers or impacts [9]. Furthermore, linking different types of models, each providing an application at a specific scale, is used to downscale macro-level findings or to describe dynamics in nested systems [10].

The overall goal of this paper is to contribute to closing the knowledge gap between micro- and macro-level understandings of the nexus between land use, poverty, and environment. It will build on “meso-scale” approaches which have been developed to generate and synthesize knowledge at an intermediate or regional level of spatial scale. At this level, macro-level external driving forces may still be detectable before disappearing in the heterogeneity of the local context, and local conditions have not been aggregated to a point where they are no longer recognizable [44–46]. In the concrete case of the present study we selected the national level of Laos as the meso-scale. In a first step we perform a spatial analysis of the interrelations between land use, poverty, and environment at landscape level for the entire territory of Laos. Analysing the resulting spatial patterns will allow us to reveal generalizable trends but also crucial exceptions. In a second step we explore if the configurations of the nexus between land use, poverty, and environment are more strongly shaped by external driving forces or by local factors. To this end, we use accessibility and ethnicity as proximate variables. Finally, we discuss what trade-offs different development pathways imply in terms of poverty alleviation and environmental degradation, and conclude on main policy implications and future research needs.

## Materials and Methods

We describe configurations of land use, poverty, and environment for the entire territory of Laos, synthesizing four data sets emerging from three previous studies:

1. Landscape mosaics as characterized by land use and environmental status [46];
2. Village-level socioeconomic data including demography and ethnicity from the Socio-Economic Atlas of the Lao PDR [47]
3. Estimates of poverty density and incidence at village level [31];
4. Accessibility expressed as travel time to nearest district capital [31].

In the following subsections, we present these four data sets as well as our approach.

### 2.1. Landscape mosaics providing information on land use and environmental status

**Fig 1:**
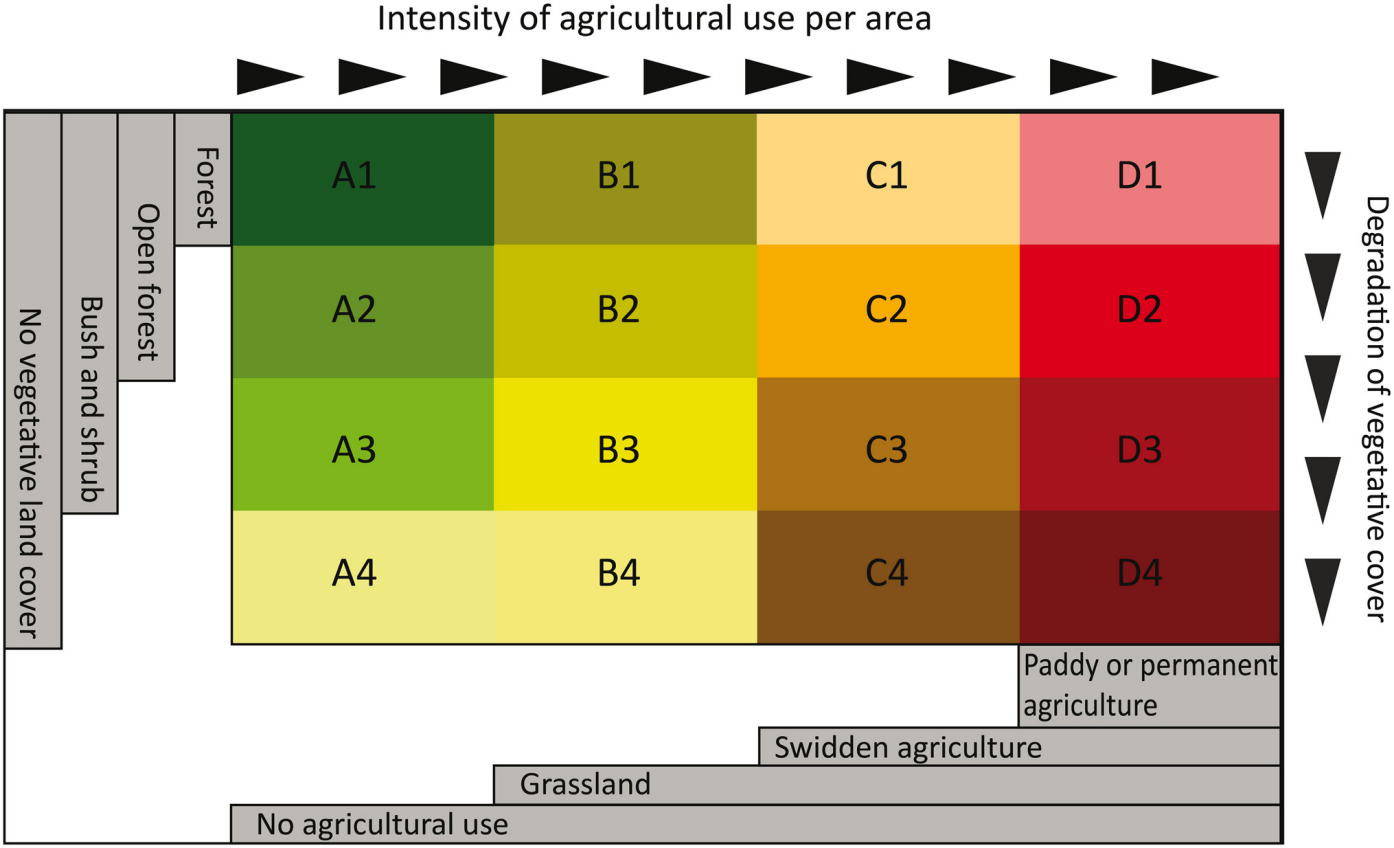
Classification of landscapes mosaics. This chart illustrates the definition of landscape mosaics by the intensity of agricultural use (columns) and the degradation of vegetative cover (rows). The letters designate land use intensity (A no agricultural use, B grassland, C swidden agriculture, and D paddy or permanent agriculture); the number designates vegetation status (1 forests, 2 open forests, 3 bush and shrub, 4 no vegetative land cover) Source: [46].

This data set emerges from previous research in Laos by Messerli et al. [46]. It describes the entire territory of 236,800 km^2^ in terms of 16 different types of landscapes, each of which represents a specific combination of two key characteristics (Fig 1): first, the intensity of agricultural use per area determining the dominant land use category (no use, grassland, swidden agriculture, paddy or permanent agriculture) and second, the least degraded form of natural vegetation (forests, open forests, bush and shrub, no vegetative land cover).

In general terms, the approach underlying these landscape mosaics is based on the insight that translating land cover information—commonly obtained from satellite imagery—into people’s actual land use, beyond the local scale, represents a persistent challenge in land science. Without knowledge about a local context we cannot determine if for example a patch of shrub represents fallow land in swidden agriculture, an agroforestry home garden, or even pasture land for livestock. Yet, if information on the surrounding land cover patches were made available, one could more easily interpret the original pixels in terms of their use. In this example, if a pixel of shrub is surrounded only by pixels showing burnt fields and forests, it would, most probably, represent fallow land in a swidden agricultural landscape.

More concretely, the approach used to produce the landscape mosaics consisted of the following steps: (i) For every land cover pixel within the country, the types of land covers pixels surrounding it were analysed (land cover maps at a resolution of 50x50 m, based on availability from the Ministry of Agriculture and Forestry, 2002 [48]). Using a moving window technology in Arc-GIS, each pixel was attributed information about the composition of its neighbouring pixels in a 5×5 km window. (ii) Adjacent pixels manifesting the same neighbourhood characteristics were then clustered into a polygon. This resulted in a total of 3,446 polygons. (iii) We then classified these compositions of land cover pixels into landscape types based on the most intensive land use occurring in the polygon (columns in Fig 1) and the least degraded form of vegetation observed in the polygon (rows in Fig 1). The 16 different types of landscapes hence represent specific trade-offs between land use intensity and the different states of vegetation of the landscape. In the biophysical context of Laos the state of vegetation represents an adequate indicator for the general status of the environment, as it closely relates to soil quality, biodiversity, water retention and regulation, and carbon sequestration capacity. [39] Finally, it is important to note that these emerging landscape types feature a genuine geometry that does not refer to pre-defined administrative or bio-physical units.

### 2.2. Village-level data on population and ethnicity

For village-level socio-economic data we drew on the Socio-Economic Atlas of the Lao PDR [47]. The Atlas contains 70 indicators calculated for each of the 10,547 villages, with data based on the Population and Housing Census of 2005 [49]. As official village boundaries do not yet exist for the whole of Laos, we used village polygons that were calculated using a concept of equal travel time between the two closest village centres [31]. These village polygons were used to depict all village-level data and to calculate local population densities. We also used ethnicity data, aggregated in the Atlas to four ethno-linguistic families, 10 ethno-linguistic categories, and 49 ethnic groups. For the purposes of this paper we further aggregate these figures into two classes, representing the ethnic majority (one ethno-linguistic family of Lao-Tai) and the ethnic minority (three ethno-linguistic families: Mon-Khmer, Sino-Tibetan, and Hmong-Mien). We will hereafter refer to these as ethnic majority or ethnic minority groups. As Laos is a multi-ethnic country where different groups differ not only linguistically but also in terms of their societal position, economic status, and history [50,51], we consider ethnicity a proximate indicator for the limitations and potentials of endogenous development in a given local context.

### 2.3. Estimates of poverty at village level

Epprecht et al. [31] estimate that 40% of people in Laos live below the national poverty line. The national poverty line corresponds to the per capita expenditure required to purchase 2,100 Kcal per person per day, including the value of home production and using a household’s food basket plus a basket of non-food items. Epprecht et al. used the “small area estimation” method, drawing on information from both the 2005 Population and Housing Census [49] and the Lao Expenditure and Consumption Survey (LECS) of 2003. While this method fails to take into account many important dimensions of poverty in Laos, it has the great advantage of providing a nationwide comparable measure of people’s subsistence levels, revealing if their per capita consumption expenditure is sufficient to cover their basic needs.

### 2.4. Accessibility as proxy for external influences and opportunities

For this study, we define accessibility as travel time between any given location and the nearest district capital, which presumably offers services such as markets, agricultural inputs, extension services, and healthcare. Accessibility is a key determinant of land use and land use changes, especially in a country such as Laos where transportation infrastructure is weak but rapidly growing [38,52,53]. Epprecht et al. [31] have developed a model to estimate travel time from any point in the country to the nearest district capital using cost–distance functions based on raster GIS data. Calculated travel time assumes the availability of the best typical means of transport, and takes into account transport infrastructure, topography, and land use. However, physical accessibility alone does not guarantee actual access to such services, which may be constrained by factors such as the socio-economic assets of a household, ethnicity, or language. In this paper we use accessibility as a rather coarse proximate indicator for potential external influences on land use (investments, policies, services) and the opportunities offered by district centres as gateways to development (marketing, information, access to services).

### 2.5. Overlays and spatial analysis

In order to describe different configurations of land use, poverty, and environment, we relate these different data sources using spatial intersects and descriptive statistics. We intersect two distinct geometries: on the one hand, the landscape polygons containing information on land use and environment; on the other, the village polygons containing the socio-economic data and average accessibilities (as average travel time to district capitals). The 3,446 landscape polygons are hence overlaid with 10,547 village polygons resulting in a total of 26,334 polygons, hereafter called intersects, each containing a full data set on land use, poverty, and environment (S1 File). In the subsequent analysis, we (i) analysed different types of landscapes in terms of population, population density, and mean poverty rate; (ii) reclassified the poverty data into two classes: villages with lower poverty rates than the national rural poverty rate (hereafter called “wealthier”) and villages with higher poverty rates (“poorer”); and (iii) calculated for each intersect mean accessibility and share of population belonging to the ethnic majority group, to assess how these proximate variables explain the configurations of land use, poverty, and environment.

## Results

### 3.1. The nexus of land use, poverty, and environment in Laos

**Fig 2:**
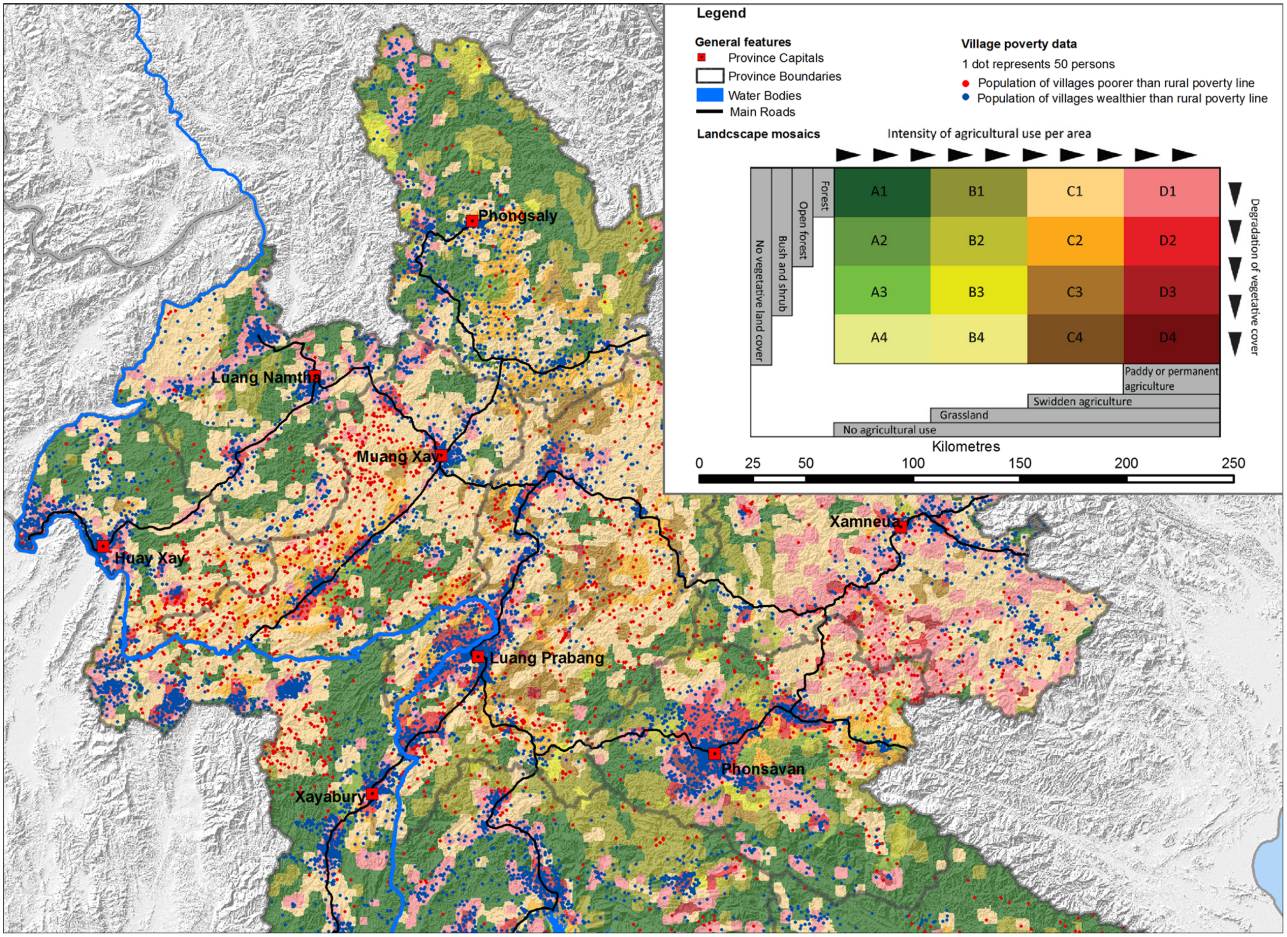
Spatial representation of the nexus between poverty, land use, and environment for northern Laos. Coloured polygons represent landscapes classified in terms of trade-offs between land use intensity and degradation of vegetation. Poverty is indicated by dot densities (1 dot = 50 persons). The total poor population in villages poorer than the national average is shown in red dots, while the total wealthier population in villages wealthier than the national average are shown in blue dots. Adapted from Messerli [46] under a CC BY license, original copyright 2009.

In Fig 2, combinations of land use, poverty, and environmental status show complex and fine-grained patterns across space. Landscapes with paddy or permanent agriculture but degraded vegetation (shaded in dark red) manifest generally high densities of comparatively wealthier populations (blue dots). Conversely, degraded swidden agricultural landscapes (shaded in orange and brown) tend to be inhabited by poorer and fewer people (red dots). Nevertheless, crucial exceptions to this trend can easily be found. Along the north-eastern road leading from Muang Xay towards Vietnam, we detect wealthier people living in swidden agriculture; around Phongsaly, at the northern tip of Laos near the Chinese border, wealthier people practice paddy or permanent agriculture but live in landscapes where forests persist (pink colour), an indication that their activities are not degrading the environment. In summary, we can observe almost all configurations of the nexus between land use, poverty, and environment, with parameters changing frequently within short distances. A high-resolution analysis such as this supports case-study-based research defending the high diversity of poverty–environment relations in rural Laos.

Table 1 shows aggregations of the different combinations of the nexus between land use, poverty, and environment at landscape level. About one-third of all landscapes in Laos are characterized by swidden agriculture and one-third by paddy or permanent agriculture. These landscapes host 17% and 74% of the total population respectively. Although the poverty rate in swidden agriculture is higher (50%) than in paddy or permanent agricultural landscapes (33%), the absolute number of poor people living in paddy or permanent agriculture is significantly higher, as their population density is greater. In other words, 60% of all poor people live in paddy or permanent agricultural landscapes.

**Table 1:** Landscape-level aggregations of land use, poverty, and environment.

				Intensity of agricultural use				
				A.	B.	C.	D.	
				No agriculture	Grassland	Swidden agriculture	Paddy or permanent agriculture	*Row totals / means*
**Degradation of vegetative cover**	**1. Forest**	Area share of Laos	[Percent]	30.9	7.0	21.8	13.9	*73*.*7*
		Population share of Laos	[Percent]	6.3	1.2	11.1	16.6	*35*.*2*
		Population density	[Pers./km2]	6.5	4.9	11.5	24.0	*11*.*7*
		Poverty rate	[Percent]	47.7	51.1	49.9	41.0	*47*.*4*
	**2. Open forest**	Area share of Laos	[Percent]	1.9	1.0	3.1	11.5	*17*.*5*
		Population share of Laos	[Percent]	1.1	0.5	3.1	31.1	*35*.*8*
		Population density	[Pers./km2]	13.4	8.9	21.5	52.3	*24*.*0*
		Poverty rate	[Percent]	47.0	45.6	46.7	36.3	*43*.*9*
	**3. Bush and shrub**	Area share of Laos	[Percent]	0.6	0.5	3.3	3.8	*8*.*2*
		Population share of Laos	[Percent]	0.2	0.2	2.7	16.3	*19*.*4*
		Population density	[Pers./km2]	9.2	8.2	17.5	61.2	*24*.*0*
		Poverty rate	[Percent]	50.7	49.4	54.7	31.9	*46*.*7*
	**4. No vegetative land cover**	Area share of Laos	[Percent]	-	-	-	0.6	*0*.*6*
		Population share of Laos	[Percent]	-	-	-	9.6	*9*.*6*
		Population density	[Pers./km2]	-	-	-	120.4	*120*.*4*
		Poverty rate	[Percent]	-	-	-	22.8	*22*.*8*
	**Column totals**	**Area share of Laos**	**Percent**	**33.4**	**8.5**	**28.3**	**29.8**	**100.0**
		**Population share of Laos**	**Percent**	**7.7**	**1.9**	**16.9**	**73.6**	**100.0**
		**Population density**	**Pers./km2**	**9.7**	**7.3**	**16.8**	**64.5**	**45.0**
		**Poverty rate**	**Percent**	**48.5**	**48.7**	**50.4**	**33.0**	**40.2**

This table provides an analysis of different combinations of the nexus between land use, poverty, and environment at landscape level. For each landscape described in terms of land use intensity and status of the vegetation, we calculated the respective shares of area and population of Laos, as well as population densities and poverty rates.

In terms of the environment, over 90% of the territory consists of landscapes with forest patches (73.7%) or degraded forests (17.5%). These landscape types host 71% of the population. Swidden agriculture occurs almost exclusively near forests and only to a small extent in degraded environments. With an increasingly degraded environment, poverty rates in swidden agriculture also increase while population densities first increase, then decrease. The major part of paddy or permanent agriculture still coexists with forests and degraded forests, hosting the largest share of the population. But paddy or permanent agriculture is also the land use that occurs most in environments with degraded vegetation: 29% of people live on 8.8% of the land area where only bush and shrub or hardly any detectable vegetation cover remain. It is interesting to note that in paddy or permanent agriculture with less intact environments, population densities increase while poverty rates sharply decrease. This contrasts with trends in swidden agriculture.

In very general terms we may conclude that paddy or permanent agriculture covers only 30% of the territory but hosts nearly 75% of the people; it has the lowest overall poverty rate but the highest trade-off with the environment. Swidden agriculture covers almost the same share of the territory, hosting 17% of the population in forested landscapes at a higher poverty rate.

### 3.2. Assessing patterns of the nexus between land use, poverty, and environment

**Fig 3:**
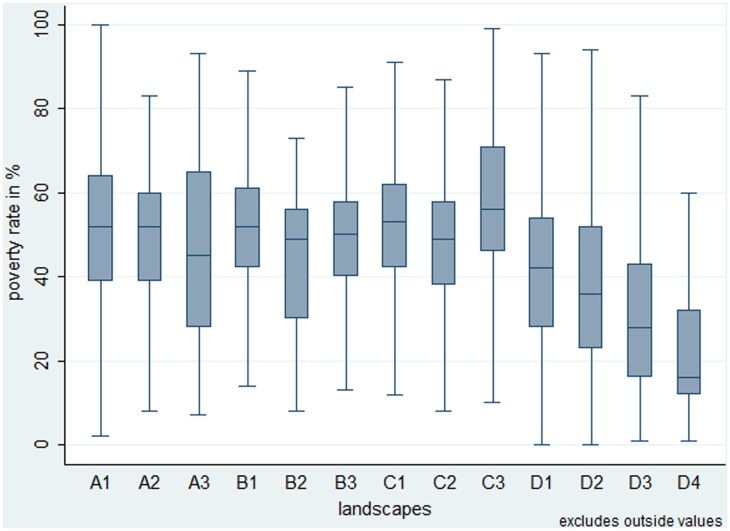
Assessing differences of poverty between landscapes. Boxplots of different landscapes showing the distribution of poverty rates. The labels of different landscapes (A1 to D4) correspond to the classification in Fig 1. The letter designates land use intensity (A no agricultural land use, B grassland, C swidden agriculture, D paddy or permanent agriculture); the number designates vegetation status (1 forests, 2 open forests, 3 bush and shrub, 4 no vegetative land cover)

Despite these first general trends identified in Table 1, the differences between landscape types and their poverty rate are statistically hardly significant. Fig 3 shows that poverty rates vary considerably within each landscape type, blurring any clear pattern. In other words, we see no statistically significant trade-off between intensification of land use, environmental degradation, and poverty alleviation. The only significant differences in terms of poverty seem to exist between high poverty in degraded swidden agriculture landscapes (C3) and low poverty in paddy or permanent agricultural landscapes that are moderately or strongly degraded (D3 and D4). Conversely, these paddy or permanent agricultural landscapes show the most significant differences in poverty rates compared to various other landscapes.

**Fig 4:**
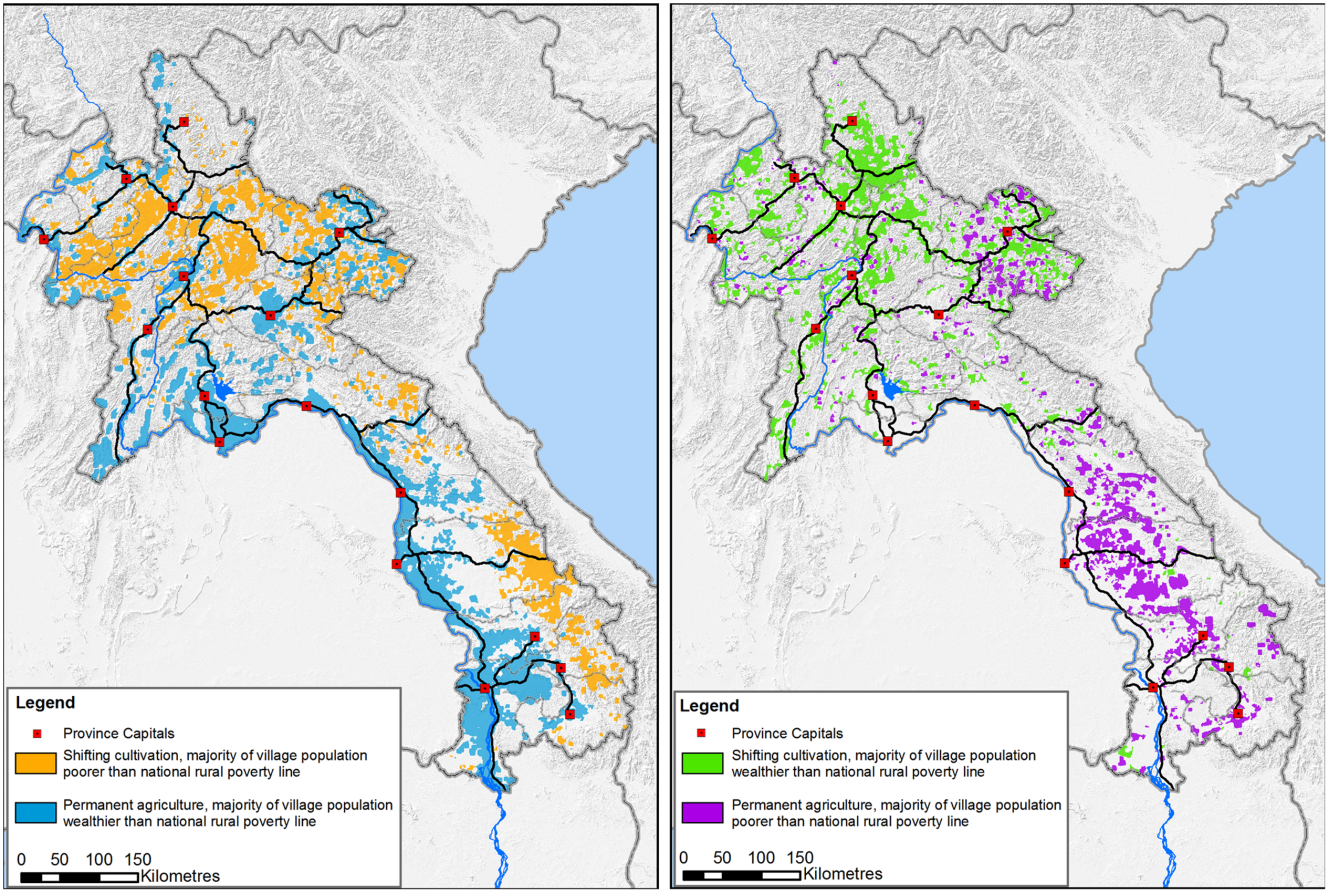
Spatial patterns of the nexus between land use intensification and poverty. The left figure seems to confirm mainstream development thinking that people in extensive swidden agriculture are poorer (orange) and people in paddy or permanent agriculture are wealthier than the average rural poverty line. Yet the figure on the right shows crucial exceptions with poorer people in paddy or permanent agriculture and wealthier people in swidden agriculture. Adapted from Messerli [46] under a CC BY license, original copyright 2009.

As poverty rates do not differ significantly when aggregated to different landscape types, we now turn towards the spatial arrangement of different configurations of land use, poverty, and environment across the country. Fig 4 focuses on the nexus between land use types and poverty, leaving the environmental dimension aside. This is because only a small share of the landscapes manifests a degraded environment (according to Table 1, 8.2% of the country has only bush and shrub, and 0.6% has no vegetative land cover left). Furthermore, poverty was reclassified into “poorer” and “wealthier” villages, depending on whether they have average poverty rates above or below the national rural average of 40%.

The left map shows poorer villages in swidden agriculture and wealthier villages practising paddy or permanent agriculture (11.7% and 53.0% of the national population respectively). This spatial pattern seems to justify the dominant development paradigm that intensifying land use will alleviate poverty. Such mainstream thinking is confirmed in the intensively cropped lowlands along the Mekong bordering Thailand, and the poor swidden agriculture areas in the remote uplands of the north and along the south-eastern border to Vietnam. However, the map on the right reveals crucial exceptions to this assumption. Landscapes with paddy or permanent agriculture but high poverty rates occur prominently in the southern hinterlands of Laos (17.7% of Lao population). At the same time, swidden agriculture landscapes whose inhabitants are wealthier than the national average occur in a fine-grained pattern in the north of Laos as well as in the easternmost province (5.1% of the national population). These maps show it is possible to reveal distinct spatial patterns, even if the linkages between intensity of land use, poverty, and environment are not statistically different. We hence conclude by hypothesizing that these configurations of local contexts cannot be understood by only analysing the mutual influences of land use, poverty, and environment. Instead, the distinct outcomes seem to be determined by other factors. Correspondingly, we will explore two additional factors: (i) accessibility, as a proximate variable for external influences, and (ii) ethnicity, as a proximate variable for potentials and limitations of endogenous development.

### 3.3. Accessibility and ethnicity as determinants of local configurations of the land use–poverty–environment nexus

Southeast Asia is in the midst of an agrarian transition that involves moving away from rural subsistence-oriented agriculture amid many parallel, extremely rapid changes [54,55]. The transition comprises processes such as agricultural intensification, integration into a market-based economy, migration, new forms of regulations governing agricultural production, and urbanization. As accessibility is a prerequisite for each of these processes, we use travel time to the nearest district capital as a proximate indicator of potential external influences.

Ethnically and linguistically, Laos is a highly diverse country. Its population is the most ethnically diverse in mainland Southeast Asia, and this diversity extends to people’s adaptive responses to their natural and social environment [47]. We therefore use ethno-linguistic families as a second proximate indicator for sociocultural dimensions of livelihoods and people’s highly different capacities on encountering different external economic and policy influences.

**Fig 5:**
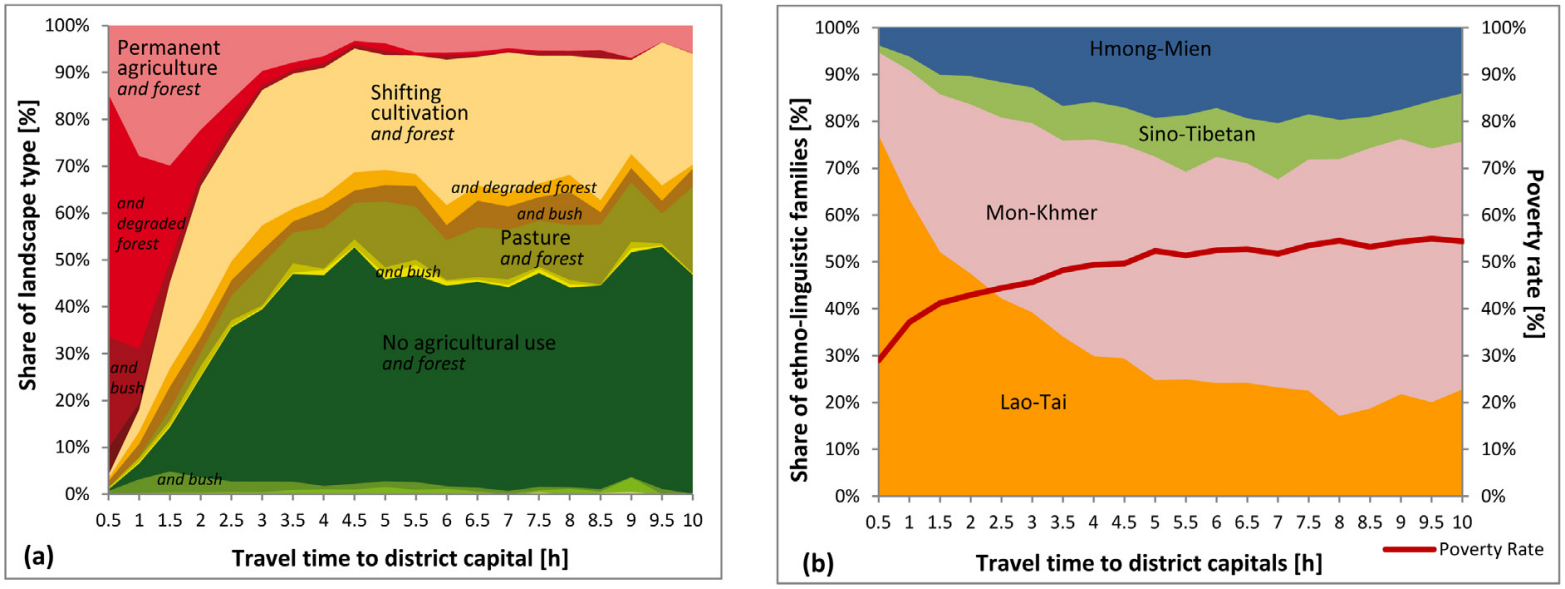
Accessibility and ethnicity as conditioning factors. Relative shares of landscape types (a) and ethno-linguistic families (b) along a gradient of accessibility, measured in travel time to the nearest district capital. Additionally, the poverty rate for each accessibility class is depicted in graph (b).

Fig 5 illustrates the proportion of landscape types (a) and ethno-linguistic families (b) along a gradient of accessibility, measured in travel time to the nearest district capital. Inaccessible parts of Laos are either dominated by swidden agriculture or other subsistence activities, or remain untouched by agricultural land use. The prevalence of forested landscape types indicates an intact natural environment. These areas are mainly inhabited by ethnic minority people suffering from high poverty rates. Within an average of approximately three hours from the nearest district capitals, we see rapid land use transitions. Forested landscapes give way to more anthropogenic environments or farm environments. In these areas the proportion of ethnic majority groups (ethno-linguistic group of Lao-Tai) is significantly higher and poverty rates drop continuously, increasing again slightly in urban centres.

These patterns reflect, to a certain degree, the place-based characteristics of the Lao territory, with ethnic minorities living in inaccessible, mountainous areas and ethnic majority people living in the more easily accessible lowlands. At the same time, the pattern depicted in Fig 5 also points to rural development processes over time. As accessibility is continuously upgraded, ethnic minority groups gain access to roads, meeting in-migrants of ethnic majority groups. In such areas, land use has been transformed into paddy or permanent agriculture at the expense of the environment. From this perspective, Fig 5 can be interpreted in terms of a space-for-time substitution, showing landscape transformations over time where inaccessible areas represent early, and accessible areas advanced stages of rural development.

**Fig 6:**
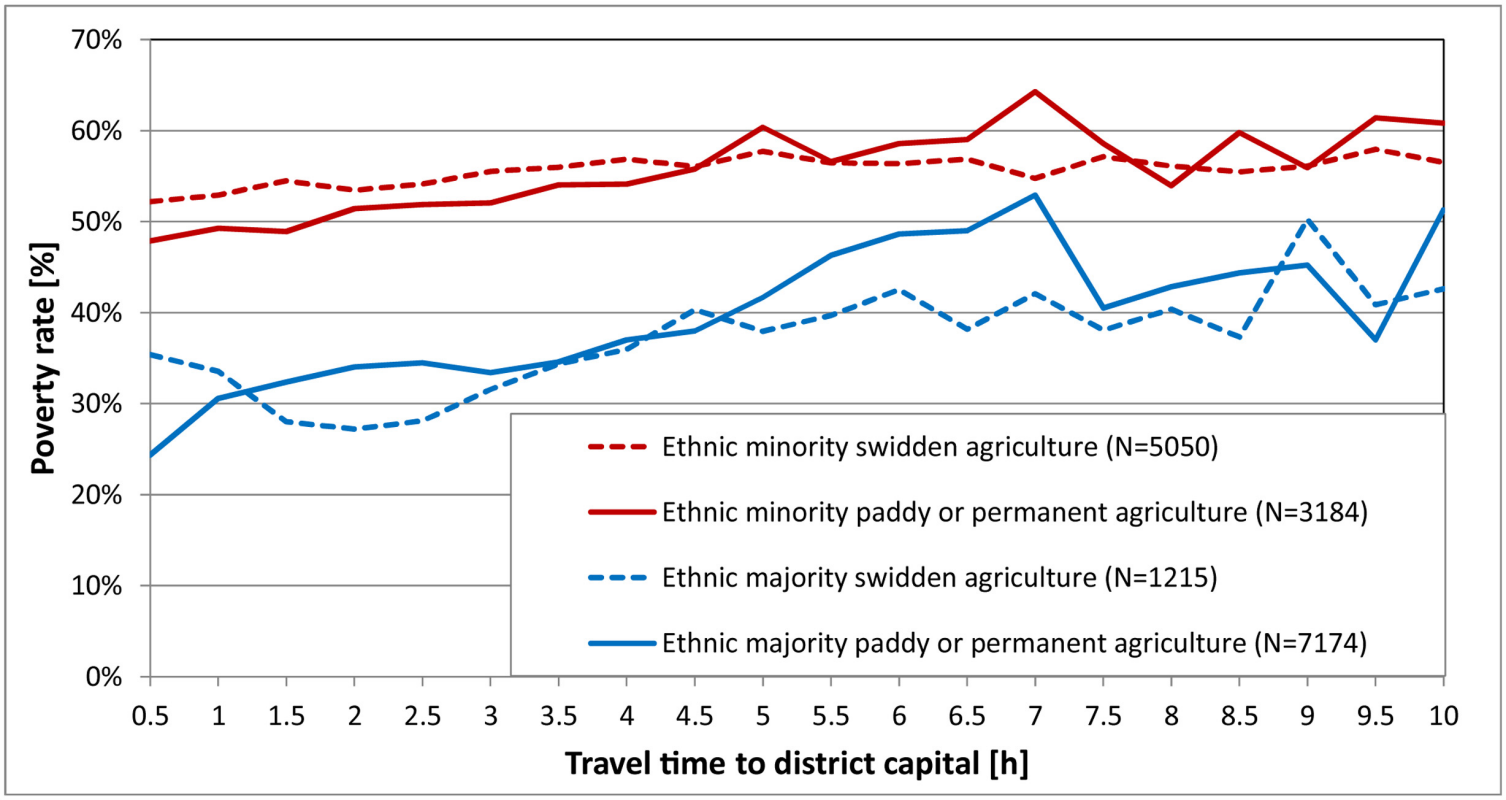
Ethnicity and accessibility as determinants of the landsape–poverty nexus. The graph shows relations between accessibility as travel time to nearest district capitals in hours, and poverty rates for four different types of village land: ethnic minority swidden agriculture, ethnic minority paddy or permanent agriculture, ethnic majority swidden agriculture, and ethnic majority paddy or permanent agriculture.

These results suggest that the nexus between land use, poverty, and environment cannot be understood independently of external driving forces and local socio-cultural factors. For this reason we wanted to further explore the interplay between these factors and the role of accessibility and ethnicity in terms of land use and poverty. We therefore analysed the relation between accessibility and poverty rates for four different types of village land: ethnic minority and ethnic majority village land in each of paddy or permanent agriculture and swidden agriculture landscapes (Fig 6). The threshold for defining ethnic minority or majority villages was set at 10% of people from the Lao-Tai ethno-linguistic family.

Fig 6 reveals three important insights. First, ethnicity has a very strong influence on the level of poverty almost independently of land use intensity and accessibility. In other words, ethnic majority groups are better off in any class of accessibility regardless of land use. Second, ethnic minority people seem to be less capable of transforming improved accessibility into reduced poverty rates. Their poverty rates—especially those of ethnic minority villages—remain largely constant across the accessibility gradient. It seems that their ethnic and linguistic background prevents accessibility from translating into access to services or development opportunities. We can thus say that the social distance between different ethnic groups outweighs the physical or geographical distance to development opportunities [31]. Third, landscapes with paddy or permanent agriculture seem to benefit more from improved accessibility, as poverty rates decrease more rapidly when approaching district capitals. This applies to land permanently cultivated by ethnic majority as well as ethnic minority groups.

## Discussion and Conclusions

The goal of this paper was to produce evidence towards closing the gap between the often generalized macro-level understandings of the land use–poverty–environment nexus and the highly contextual and diverse evidence at micro-level. We chose a meso-scale approach to analyse key indicators for the territory of Laos. In the following section we discuss our insights in light of earlier studies presented in section 1. This will allow us to reflect on implications for sustainable development oriented policies on the one hand, and future research in land system science on the other.

### 4.1. Key findings of the study

One of the guiding questions of this research was to identify whether there is a nexus between land use, poverty, and environment in Laos, as hypothesized by various studies mentioned. The descriptive analysis of 26,334 spatial polygons providing information on the intensity of land use types, environmental status, and poverty rates revealed general trends that seem to confirm, to a certain degree, mutually reinforcing linkages among these variables. One-third of the territory is dominated by extensive swidden agriculture with about 17% of the population living in relatively high poverty. The few areas of advanced environmental degradation in swidden agriculture correlate with elevated poverty rates. Landscapes dominated by paddy or permanent agriculture occupy about one-third of the territory and are home to almost 75% of the people living in comparatively lower poverty. In areas where population density rises above 60 inhabitants per km^2^, natural vegetation decreases and poverty rates decrease as well. Based on these findings, we might conclude that intensifying land use allows more people to live in less poverty, but comes at the expense of the environment by decreasing natural vegetation. Yet, we have clearly shown that most of the differences between these landscape characteristics are not significant, and hence we cannot stipulate a simple poverty–environment nexus. In fact, considerable evidence pointed to an inverted relationship, showing prevailing poverty in paddy or permanent agriculture landscapes and considerable welfare in swidden agriculture landscapes. These counterintuitive findings would lend themselves to a contrasting hypothesis: in many parts of the country people living off swidden agriculture and in an intact environment are less vulnerable and hence less poor than people in intensified and highly anthropogenic landscapes.

The spatial analysis of these contradicting hypotheses revealed interesting patterns. The distinct configurations of land use, poverty, and environment across the territory appear not to be arbitrary as statistical analysis suggests, but conditioned by factors previously not considered. For this reason we further explored accessibility and ethnicity as additional determinants. Our analysis showed how the concurrence of these factors shapes the outcomes of the land use–poverty–environment nexus. Ethnicity, considered an indicator of endogenous development potential, strongly influences poverty in all types of land use, almost independently of accessibility. Social distance hence seems to outweigh geographic or physical distance [31]. In turn, accessibility—almost a necessary precondition for lower poverty rates—is mainly useful for ethnic majority groups and people in paddy or permanent agriculture. These groups are in a better position to translate accessibility into access to services and markets and ultimately into poverty alleviation—a phenomenon revealed by various previous studies [34,41,52,53].

We are aware that research in a highly dynamic context such as Laos risks becoming rapidly outdated. For one, we observe new developments that considerably influence the configurations of land use, poverty, and environment, such as the expansion of large-scale land acquisitions [37,43], initiatives to reduce emissions from deforestation and forest degradation (REDD) [56,57], and changing land laws and policies [58]. For another, new data layers are emerging that will enable more differentiated characterizations of land use as well as of human well-being. Nevertheless, we believe that the following insights will continue to be relevant for future studies. Searching for a nexus between land use, poverty, and environment in order to inform policy is irrelevant at best or misleading at worst if we narrow our analytical lens solely to the interactions among these three factors. Instead, we should consider the various configurations of the nexus as an outcome of how, in an increasingly globalized world, these factors interact with endogenous development potentials and exogenous driving forces.

### 4.2. Implications for policy

These insights point to a twofold challenge for interventions and policies aimed at maximizing synergies and minimizing trade-offs between human well-being and environmental stewardship. First, such policies need to address the intensive cross-sectoral dynamics between land use, poverty, and environment. While this is not new and integrated conservation and development projects known as ICDPs emerged widely in the 1990s, their contested performance in conjunction with the orientation on Millennium Development Goals has led to a re-emergence of sectoral and short-term, results-oriented interventions, or market-based approaches such as payment for ecosystem services schemes, or PES. Second, our findings suggest that direct interventions on agricultural intensification, poverty alleviation, or environmental stewardship might not yield the expected results. Given the strong influence on the configuration of these factors by other variables such as ethnicity and accessibility, we suggest in more general terms that the main leverage points lie in the way external drivers meet local and highly contextual development potentials. In other words, poverty and environment outcomes could be more effectively influenced if bundles of external influences—such as foreign direct investments, market opportunities, development assistance, or public policies—can be combined to respond to the diverse endogenous development potentials of different local contexts. Strategically, this would imply not only an improved cross-sectoral coordination of development interventions, but also spatially differentiated and hence decentralized development approaches. We believe this applies not only to the specific rural development challenges in Laos, but also more generally to the land-based management of sustainability trade-offs.

### 4.3. Implications for future research

With regard to research, this study intended to demonstrate how a meso-scale approach can contribute to overcoming the dichotomy and even contradictions between macro-level (generalized but policy relevant) and micro-level findings (in-depth contextual knowledge but limited validity across scales and locations). We learned that a meso-scale approach can succeed if the analytical focus is not narrowly set to pre-determined indicators at a fixed scale. This requires choosing scale and resolution according to the development issue at stake, and balancing external driving forces with local and place-based characteristics. However, we must keep in mind that this type of analysis only provides correlations between selected indicators, and sheds no light on processes and causal interactions in the land use–poverty–environment nexus and their conditioning factors. It thus remains unclear, for example, when and how foreign direct investment into agriculture leads to new forms of poverty or to improved well-being. Land-system science hence faces the challenge of linking patterns of quantitative and place-based indicators to insights from process-based and often qualitative studies on drivers of land use change [59,60]. Such insights would not only greatly improve our understanding of increasingly complex, cross-scale, and distant interactions between land resources and different actors; it would also significantly improve the evidence base for navigating development trade-offs between planetary boundaries and social foundations of human development.

## Supporting Information

S1 File Supplementary data on the nexus between land use, poverty and environment. This Excel file contains different variables analyzed in this paper regarding land use, poverty, and environment for 26,334 spatial polygons representing the intersects between village areas and landscape types.
(XLSX)
